# Characterization of the chemoreceptor repertoire of a highly specialized fly with comparisons to other *Drosophila* species

**DOI:** 10.1590/1678-4685-GMB-2022-0383

**Published:** 2024-06-14

**Authors:** Pedro Mesquita Fonseca, Lizandra Jaqueline Robe, Tuane Letícia Carvalho, Elgion Lucio Silva Loreto

**Affiliations:** 1 Universidade Federal do Rio Grande do Sul, Instituto de Biociências, Programa de Pós-Graduação em Genética e Biologia Molecular, Porto Alegre, RS, Brazil.; 2 Universidade Federal de Santa Maria, Centro de Ciências Naturais e Exatas, Programa de Pós-Graduação em Biodiversidade Animal, Santa Maria, RS, Brazil.; 3 Universidade Federal de Santa Maria, Centro de Ciências Naturais e Exatas, Departamento de Bioquímica e Biologia Molecular, Santa Maria, RS, Brazil.

**Keywords:** Olfaction, taste, odorant receptor, gustatory receptor

## Abstract

To explore the diversity of scenarios in nature, animals have evolved tools to interact with different environmental conditions. Chemoreceptors are an important interface component and among them, olfactory receptors (ORs) and gustatory receptors (GRs) can be used to find food and detect healthy resources. *Drosophila* is a model organism in many scientific fields, in part due to the diversity of species and niches they occupy. The contrast between generalists and specialists *Drosophila* species provides an important model for studying the evolution of chemoreception. Here, we compare the repertoire of chemoreceptors of different species of *Drosophila* with that of *D. incompta*, a highly specialized species whose ecology is restricted to *Cestrum* flowers, after reporting the preferences of *D. incompta* to the odor of *Cestrum* flowers in olfactory tests. We found evidence that the chemoreceptor repertoire in *D. incompta* is smaller than that presented by species in the *Sophophora* subgenus. Similar patterns were found in other non-*Sophophora* species, suggesting the presence of underlying phylogenetic trends. Nevertheless, we also found autapomorphic gene losses and detected some genes that appear to be under positive selection in *D. incompta,* suggesting that the specific lifestyle of these flies may have shaped the evolution of individual genes in each of these gene families.

## Introduction

Nature consists of many environments with different conditions and particularities. Animals living in each environment are in constant contact with many chemical compounds. Some of these compounds come from other living forms and are received and interpreted for many different purposes, such as finding sexual partners (Wyatt, 2003; Davis, 2007; Kurtovic et al., 2007), identifying predators or parasites (Ebrahim et al., 2015; Joseph and Carlson, 2015), or even ﬁnding and tasting food (Dahanukar et al., 2005; Hallem et al., 2006). Regardless of the nature of the chemical signals, animals usually interpret them through chemoreceptors present in their cells. Because chemoreceptors are directly involved in this first contact with nature, they are subject to differential selection pressures during animal evolution (Whiteman and Pierce, 2008). In this sense, chemoreceptor genes generally evolve fast, leading to great diversification in genic repertoire (McBride, 2007; McBride and Arguello, 2007; Vieira and Rozas, 2011; Cande et al., 2013). These patterns generally lead to an association between odor detection and individual ecological needs (Carey et al., 2010).

Since the early 20th century, many *Drosophila* species have been used as model organisms in various scientific fields, among which we can highlight *Drosophila melanogaster* Meigen, 1830 (Markow, 2015). Nevertheless, the genus includes 1,644 described species with many different traits, niches, and distributions (O’Grady and DeSalle, 2018; TaxoDros). Many of these species have provided excellent models for evolutionary genomics, and there is a wealth of phylogenetic information spanning hundreds of species and at least thirty well-annotated genomes (Drosophila 12 Genomes Consortium *et al.*, 2007; Chen et al., 2014; O’Grady and DeSalle, 2018). *Drosophila* also provides several advantages while studying adaptations associated with different ecological habits, as it includes species with contrasting behaviors and niche breaths. Among these species we can identify generalists such as *D. melanogaster*, *Drosophila ananassae* Doleschall (1858), and *Drosophila simulans* Sturtevant (1919) and other specialists such as *Drosophila grimshawi* Oldenberg, 1914, *Drosophila erecta* Tsacas and Lachaise (1974) and *Drosophila sechellia* Tsacas and Bächli, 1981. Generalist habits allow exploring a variety of resources for feeding and oviposition, making such species resilient under conditions of limited resource availability or competition (Anholt, 2020). Conversely, specialists benefit from low competition for food or oviposition sites once they can overcome toxic or extreme niches (Anholt, 2020).

Among the different families of chemoreceptor genes, those coding for gustatory receptors (GR) and olfactory receptors (OR) are responsible for the detection of flavors or odorants, respectively (Clyne et al., 1999; Gao and Chess, 1999; Scott et al., 2001; Vosshall and Stocker, 2007; Martin et al., 2013). In *D. melanogaster*, each of these two gene families harbors 60 genes (Robertson et al., 2003). These genes are commonly involved in adaptations to different ecological contexts (Depetris-Chauvin et al., 2015; Diaz et al., 2018). Such a pattern has been demonstrated, for example, for the pestiferous species *Drosophila suzukii* Matsumura that differs from other species of the *D. melanogaster* group due to its characteristic of exploring fresh fruit rather than rotting ones, which is reflected in the OR and GR repertoire (Hickner et al., 2016). Moreover, specialist *Drosophila* species can present a fivefold higher dN/dS ratio for OR and GR genes (McBride, 2007) and may also present faster rates of gene loss for these two gene families (McBride and Arguello, 2007). Nevertheless, according to Gardiner et al. (2008), endemism rather than niche specialization may account for much of this straightforward chemosensory gene loss. 

Within *Drosophila*, the *D. flavopilosa* species group draws attention for its close association with flowers of *Cestrum* (Solanaceae), using this substrate as a unique resource for oviposition, larval development, and adult feeding (Brncic, 1966; Robe et al., 2013). This group consists of 18 species (TaxoDros) divided into two subgroups and is considered part of the *virilis-repleta* radiation (Throckmorton, 1975), which is currently considered a subgenus of the *Drosophila* genus (the subgenus *Siphlodora*) (Yassin, 2013). Nevertheless, the exact phylogenetic position of the group within the subgenus remains controversial, as it can be considered either a sister group of the *D. annulimana* species group (Robe *et al.*, 2010) or a sister group of the *D. virilis* or *D. repleta* species groups (de Ré *et al.*, 2017). The flies of the *flavopilosa* group share some strict morphological and physiological adaptations for the use of *Cestrum* flowers: the pale yellow color of the body, which is cryptic in many *Cestrum* flowers; the medium to small size of the flies; oviposition at advanced stages of embryonic development, which matches with the ephemeral nature of *Cestrum* flowers; and the robust and full sting characteristic of ovipositors used to scarify the flower surface (Wheeler et al., 1962; Pipkin et al., 1966; Brncic, 1983; Ludwig et al., 2002; Robe *et al.*, 2013). Although the occurrence of this group covers a large portion of the Neotropical region, most species are endemic to small patches (Robe *et al.*, 2013). The most widespread species in the group is *D. incompta*, which occurs from Argentina to Mexico (TaxoDros).

The large number of chemoreceptors that can be found in *Drosophila* possibly enables the exploration of different resources, providing means to the establishment of different niches. To relate the preference of a species to its specific resource, choice tests are usually performed comparing different substrates. In these tests, *Drosophila* usually prefers citric substrates for oviposition (Dweck et al., 2013), but this choice can vary depending on the niche of each species. Mansourian et al. (2018) demonstrated that *D. melanogaster* specimens are seasonal specialist of marula fruit (*Sclerocarya birrea*), preferring volatile marula odors over orange scents, although they preferred orange when tested with other fruit scents such as banana. Host switching between populations can also be assessed by comparing preferences for different odors. For example, for *D. mojavensis*, a cactophilic species that feeds on and breeds on various cactus species, population-specific preferences were previously detected (Date et al., 2013).

The aim of this study is to contribute to the knowledge of the patterns of molecular evolution associated with the chemoreceptor repertoire in different ecological contexts. To this end, we first examined the olfactory preferences of *D. incompta* to test if there are preferences for *Cestrum* flowers extracts over other odors. We then listed OR and GR genes that are part of the chemoreceptor repertoire in this species and compared them to homologous sequences in other *Drosophila* species to gain insight into the forces that might be shaping the evolution of chemoreceptor genes in specialized species.

## Material and Methods

### Sampling

Specimens of the *Drosophila flavopilosa* group were obtained from flowers of *Cestrum strigilatum* (Solanaceae) collected in the city of Santa Maria, southern Brazil (-29.710553 latitude and -53.717070 longitude). The sampled flowers were kept in the laboratory until the adult flies emerged. *Drosophila incompta* was distinguished from the other species of the group by its external morphology and male genitalia patterns, according to Wheeler et al. (1962).

### Olfactory choice tests

To test the flies’ preference for different odors, we performed an olfactory choice test (Table S1) with adult flies of *D. incompta* collected from the field, as described above. The tests were also performed with adults of *D. melanogaster* from the Oregon-R strain maintained in our laboratory, as a control to test if the olfactometer was working well. Flies were tested up to two days after emergence, until the total number of 10 flies was achieved per test. The elected odors were: 1) extract of *Cestrum strigilatum* L. flowers [1 ml of a solution of non-rotting flowers, prepared with five entire flowers (approximately 5 g) macerated in 5 ml of distilled water]; 2) extract of *Brunfelsia uniflora* (pohl) (Solanaceae) [1 ml of a solution of non-rotting flowers, prepared with two entire flowers (approximately 5 g) macerated in 5 ml of distilled water]; 3) extract of banana fruits (1 ml of a solution prepared with 5 g of a mature banana macerated in 5 ml of distilled water); and 4) extract of orange fruits (1 ml of a solution prepared with 5 g of a mature orange with peel macerated in 5 ml of distilled water). These odors were chosen considering the putatively strict adaptation of *D. incompta* to *Cestrum spp.* (Hofmann and Napp, 1984; Santos and Vilela, 2005), against the general profile of exploring different fermenting fruits presented by frugivorous *Drosophila* species (Sevenster and Van Alphen, 1993; Markow, 2015). Conversely, *Brunfelsia* flowers were elected because they blossom concomitantly with *Cestrum* and have records of interaction with other *Drosophila* species (Frota-Pessoa, 1952; Malogolowkin, 1953; Sepel et al., 2000; Schmitz and Valente, 2019). 

**Figure 1 -:**
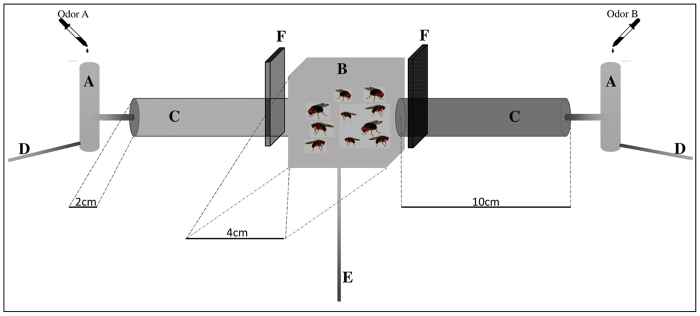
Olfactometer model used to perform the olfactory preference tests. Legend: **A**, Extracts scent vessels; **B**, Flies waiting box; **C**, Preference tubes; **D**, Air inlet pipes; **E**, Air suction pipe; and **F**, Gates separating the flies from scents.

In each case, the extracts were combined in pairs and dropped onto a new piece of cotton (Figure 1). Ten flies of the same sex were placed in the center of the olfactometer (following Fuyama, 1976) with the gates closed for two minutes for acclimation. After, gates were opened, and flies were allowed to choose a side. After five minutes, flies located on each side of the artifact were counted. All tests were conducted in a dark room with only red light available, in the morning. Each test was repeated at least six times per treatment, considering replicates performed either for males or with females, as both sexes showed similar patterns. Analysis of variance (ANOVA) was performed to assess the significance of the preferences.

### Molecular biology procedures

Total DNA was isolated from 20 males of *D. incompta* using a NucleoSpin Tissue XS kit (Macherey-Nagel, Düren, Germany) according to the manufacturer’s protocol. Only males were employed at this stage due to the cryptic nature of *D. incompta* regarding other closed related species, which hampers females’ identification (Wheeler et al., 1962; Robe et al., 2013). Two approaches were used to obtain shotgun sequences of the whole genome. First, 100-bp single-end reads were obtained using a Solexa-Illumina HiSeq 2000 Next Generation Sequencing (NGS) instrument (Fasteris DNA Sequencing Service). Furthermore, we also generated 100-bp paired end reads in a Solexa-Illumina HiSeq 2000 instrument (Illumina Inc., San Diego, USA) at Macrogen Sequencing Service (Korea) using the provider’s protocols.

Other 20 adult males of *D. incompta* were directly collected from the medium with sampled flowers up to two hours after eclosion and further employed to isolate total adult RNA using TRIzol reagent (ThermoFisher Scientific), according to the protocol of Rio et al. (2010). The mRNA was isolated using the mRNA Dynabeads C kit (Life Technologies) and libraries were prepared using the Seq RNA Total Ion V2 kit (ThermoFisher Scientific). Sequencing was performed using a Ion S5 sequencer (ThermoFisher Scientific).

The genome of *D. incompta* was assembled using a hybrid assembly approach for both paired-end and single-end libraries in hybridSPAdes (Antipov et al., 2016). The performance of the de novo assembly was evaluated using Quast (Gurevich et al., 2013) and Busco 5.4.6 software (Simão et al., 2015). In the last case, the Diptera OrthoDBv.10 (odb10) dataset was employed, with default parameters (Table S2). Transcriptome de novo assembly was performed using Trinity (Grabherr et al., 2011) under default parameters in the Galaxy platform (Afgan et al., 2018).

Predicted coding sequences (cds) and protein genes for both genome and transcriptome drafts were identified using the Augustus 3.4.0 software (Stanke et al., 2006). Both cds datasets were merged, and redundant sequences were removed using the cd-hit-2d program (Fu et al., 2012). This resulted in a non-redundant hybrid annotation of predicted cds, including genome and transcriptome sequences. 

### Recovery of OR and GR gene sequences in *D. incompta*

To retrieve sequences related to OR and GR chemoreceptors in the genome of *D. incompta*, at first, the repertory of olfactory and gustatory receptor genes of *D. melanogaster* (Robertson et al., 2003) and *D. virilis* (Drosophila 12 Genomes Consortium *et al.*, 2007) were downloaded from FlyBase (Thurmond et al., 2019), totaling 121 and 103 different genes sequences, respectively. *Drosophila melanogaster* was chosen because it is a model organism that has a well-annotated and well-studied repertoire of chemoreceptors. In turn, *D. virilis* entered as a query in our searches because it is closely related to *D. incompta* (Robe *et al.*, 2010; De Ré et al., 2017). These sequences were arranged into two separate matrices subdivided by chemoreceptor gene family. 

The pipeline employed in the InsectOR tool (Karpe et al., 2021) was then used to predict and validate OR and GR sequences in *D. incompta* genome. For this task, Exonerate was first used to align the protein sequences recovered for *D. melanogaster* and *D. virilis* against the draft genome of *D. incompta* assembled by HybridSpades. These alignment files, the draft genome, and the protein queries were then submitted as input to the InsectOR web server. As a result, detailed information on each gene prediction and cds and protein fasta sequences were obtained. Finally, for an exhaustive approach, three additional strategies were employed: 1) OR and GR gene sequences that were not found with InsectOR were searched against the Augustus annotation using protein sequences of *D. melanogaster* and *D. virilis* as queries in BLASTp and tBLASTn searches; 2) these sequences were also used as seed against the raw reads of *D. incompta* genome and transcriptome, through the use of aTRAM 2.0 software (Allen et al., 2018), which simultaneously searches and assembles orthologous gene sequences; 3) eggNOG-mapper (Huerta-Cepas et al., 2017) was employed to functionally annotate the entire genome of *D. incompta*, using the paired-end Solexa-Illumina raw reads with default parameters.

### Repertoire of OR and GR gene sequences in other Drosophila species

To recover OR and GR gene sequences from other species, we relied on the repertoires found by Gardiner et al. (2008) for the first 12 *Drosophila* sequenced genomes (Drosophila 12 Genomes Consortium *et al.*, 2007) (*D. ananassae, D. erecta, D. grimshawi, D. mojavensis, D. melanogaster, D. persimilis, D. pseudoobscura, D. sechellia, D. simulans, D. virilis, D. willistoni*, and *D. yakuba*). To complement the matrices and provide a broader glimpse about species with different levels of niche specialization, we also retrieved OR and GR genes from the genomes of other 19 *Drosophila* species (*D. arizonae, D. biarmipes, D. bipectinata, D. busckii, D. buzzatii, D. elegans, D. eugracilis, D. ficusphila, D. hydei, D. kikkawai, D. mauritiana, D. miranda, D. navojoa, D. novamexicana, D. obscura, D. rhopaloa, D. serrate, D. suzukii*, and *D. takahashii*) and outgroups (see below). These searches were performed through BLASTn in the NCBI database using OR and GR genes of *D. virilis* and *D. melanogaster* as queries. In each case, only the main copy of each gene was considered, and duplicated copies were not counted. Conversely, incomplete copies were eventually included in further analyses if they were the only available copies of the respective genes. We considered this approach a strategy to uncover the general evolutionary pattern of the two different gene families since it is not strongly affected by recent duplications or gene losses in other *Drosophila* species. Moreover, this approach is more robust to problems related to sequencing or assembling strategies.

### Orthology assignments

To correctly assign orthologous relationships, the whole set of amino acid sequences of OR and GR gene families isolated for *D. melanogaster* and *D. virilis* were aligned against the putative orthologous copies retrieved for *D. incompta*. This alignment was performed under a PAM matrix, using the Clustal W algorithm (Thompson et al., 1994), as available in Mega 7.0.26 software (Kumar et al., 2018). OR and GR matrixes were then employed to reconstruct a phylogenetic tree, under maximum likelihood (ML) in the IQTREE v.1.6.12 software (Nguyen et al., 2015), using 10,000 ultra-fast bootstrap replicates. The ML tree was finally visualized in FigTree v1.4 (2018) and rooted at the midpoint.

### Evolutionary tests

For each individual gene, matrices were constructed containing all homologous sequences found in the different *Drosophila* species and outgroups (Table S3). *Scaptodrosophila lebanonensis* was generally used as an outgroup except in cases where no orthologous sequences were found for this species. In these cases, another species closely related to Drosophilidae for which orthologous sequences were available in NCBI was used as the outgroup: *Calliphora stygia*, *Ctenopseustis obliquana,* or *Rhopalosiphum maidis* for ORs; and *Bactrocera latifrons, Musca domestica*, or *Ceratitis capitata* for GRs. Codon-based alignments were then performed individually for each gene of the GR or OR gene families for which orthologous sequences of at least 441 bp were found for *D. incompta* using MACSE (Ranwez et al., 2011).

Subsequently, two different selection tests were performed to evaluate the presence of negative or positive selection in the *OR* and *GR* genes of *D. incompta*: A) the adaptive branch-site REL test for episodic diversification (aBSREL; Smith et al., 2015) was performed to test whether the branch leading to *D. incompta* evolved under positive selection; and B) the fixed effects likelihood test (FEL; Pond and Frost, 2005) was used to derive the nonsynonymous substitution (α) and synonymous substitution (β) rates, testing whether alpha is significantly higher than beta at each site in the sequence of *D. incompta*. The latter test was applied only to matrices that showed a signal of positive selection for *D. incompta* genes in the aBSREL test. In both cases, individual gene codon alignments were submitted to the Datamonkey web platform (Weaver et al., 2018), and analysis were performed with default parameters. 

## Results

### Olfactory preference tests

**Figure 2 -:**
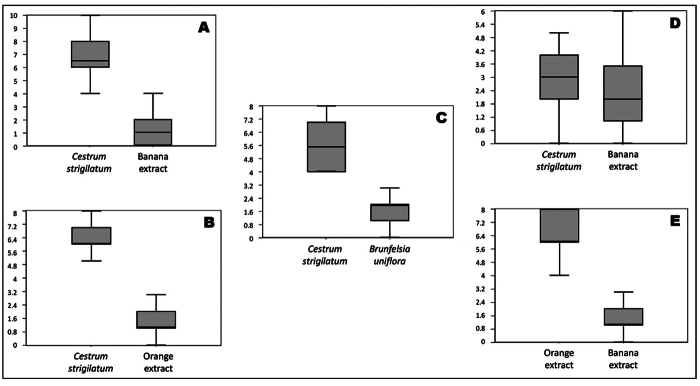
Results of ANOVAs performed to test the preference of *D. incompta* (**A**, **B**, and **C**) and *D. melanogaster* (**D** and **E**) for each pair of extract scents. **A** and **D**: *Cestrum strigilatum* against banana*. F-Stat* = 267.0286, *p* = 1.4772e-22 and *F-Stat* = 0.03866, *p* = 0.84555, respectively; **B**: *C. strigilatum* against orange*. F-Stat* = 80.2002, *p* = 1.246e-7; **C**: *C. strigilatum* against *Brunfelsia uniflora. F-Stat* = 25.71407, *p* = 0.0005; **E**: Orange against banana*. F-Stat* = 74.88571, *p* = 1.9737e-7. **Y**: axis represents the percentage of the preference of flies for both compared extracts at every paired comparison.

Despite exploring a limited variety of odors, the olfactory preference tests showed a clear preference of *D. incompta* for *Cestrum* flowers compared to fermented banana extract (*p* < 0.0001). *Drosophila incompta* also preferred *Cestrum* flowers compared to fermented orange extract (*p* < 0.0001) and *Brunfelsia uniflora* (Pohl.) flowers (*p* = 0.0005). For *D. melanogaster,* the test of preference for *Cestrum* versus fermented banana extract showed no statistically significant difference. On the other hand, our results corroborated the results found by Mansourian et al. (2018) about the significant preference of *D. melanogaster* for orange over banana extracts (Figure 2, Table S1), showing that our olfactometer provides an accurate description of smell perception and preference for the tested extracts in the target species.

### Genome-wide analyses

The *de novo* genome assembly on hybridSPAdes resulted in 187,091 contigs from 289,083,143 raw reads (including both, single and paired end sequences), which resulted in 2,437 matches in Busco (Table S2). This was contrasted by the 11,209 contigs recovered by Trinity for the transcriptome. The hybrid dataset including genome and transcriptome nonredundant cds recovered by Augustus resulted in 2,660 matches in Busco (Table S2).

### Repertoire of genes related to OR and GR gene families

After confirming orthology in a ML tree reconstructed for each family (Figs. 1S and 2S), we recovered a total of 84 sequences related to different chemoreceptors genes in *D. incompta*, of which 40 and 44 were identified as GRs and ORs, respectively (Tables 1 and 2). For ORs, 42 were recovered by InsectOR, and 11 and 37 were found by similarity searches using *D. melanogaster* and *D. virilis* genes as queries, respectively (Table S4). For GRs, all 40 genes were recovered by InsectOR, whereas 6 and 37 were found by similarity searches using *D. melanogaster* and *D. virilis* genes as queries, respectively (Table S4). Among the genes not recovered by InsectOR, EggNog mapper and aTRAM were able to recover incomplete copies of two ORs.

**Table 1 -:** Number of olfactory receptors (OR) and gustatory receptors (GR) related genes retrieved for *D. incompta* and each of the other 30 *Drosophila* species.

Species	Number of OR	Number of GR
*D. sechellia*	60	60
*D. simulans*	60	60
*D. mauritiana*	58	57
*D. melanogaster*	60	60
*D. erecta*	59	56
*D. yakuba*	59	59
*D. biarmipes*	57	55
*D. suzukii*	58	55
*D. takahashii*	57	56
*D. eugracilis*	57	56
*D. elegans*	57	52
*D. rhopaloa*	57	55
*D. ficusphila*	57	51
*D. kikkawaii*	58	51
*D. serrata*	58	51
*D. anananassea*	58	54
*D. bipectinata*	54	47
*D. persimilis*	58	52
*D. pseudoobscura*	58	52
*D. miranda*	58	50
*D. obscura*	56	48
*D. willistoni*	48	47
*D. mojavensis*	49	43
*D. navojoa*	45	40
*D. arizonae*	43	38
*D. hydei*	41	38
***D. incompta***	**44**	**40**
*D. novamexicana*	46	38
*D. virilis*	50	40
*D. grimshawi*	46	40
*D. busckii*	38	34

Note - For each species, the total number of orthologous sequences was considered, disregarding duplications. *Drosophila melanogaster*, *D. simulans*, *D. sechellia*, *D. yakuba*, *D. erecta, D. ananassae*, *D. pseudoobscura*, *D. persimilis*, *D. mojavensis*, *D. virilis*, and *D. grimshawi* had their total numbers of *OR* and *GR* based on previous works (Guo and Kim, 2007; Nozawa and Nei, 2007; Gardiner et al., 2008) in addition to homology searches.

**Table 2 -:** Repertoire of olfactory receptors (OR) and gustatory receptors (GR) found in D. incompta, with their respective CDS fragment size and evidence of positive selection.

*OR*	**CDS Fragment size recovered for *D. incompta* (bp)**	**Signals of positive selection for *D. incompta* according to aBSREL (*p* ≤ 0.05)**	**Number of sites with evidence of positive selection according to FEL (*p* < 0.05)**	*GR*	**CDS Fragment size recovered for *D. incompta* (bp)**	**Signals of positive selection for *D. incompta* according to aBSREL (*p* ≤ 0.05)**	**Number of sites with evidence of positive selection according to FEL (*p* < 0.1)**
*Or2a*	876	-	-	*Gr2a*	1074 *	-	-
*Or9a*	1152 *	-	-	*Gr5a*	648	-	-
*Or10a*	1212 *	-	-	*Gr8a*	690	-	-
*Or13a*	678	-	-	*Gr9a*	1092 *	-	-
*Or19a*	1092	Yes (*p* = 0.0005)	5 (*p* < 0.0450)	*Gr10a*	1080	-	-
*Or22c*	1050	-	-	*Gr21a*	1113 *	-	-
*Or23a*	834	-	-	*Gr22e*	1050	-	-
*Or24a*	1057	-	-	*Gr23a*	778	-	-
*Or30a*	600	-	-	*Gr28a*	753	-	-
*Or33c*	1146 *	-	-	*Gr28b*	564	-	-
*Or35a*	1227 *	-	-	*Gr32a*	637	-	-
*Or42a*	1158 *	Yes (*p* = 0.0067)	8 (*p* < 0.0402)	*Gr33a*	1442 *	-	-
*Or42b*	1194 *	-	-	*Gr39a*	609	-	-
*Or43a*	1119	-	-	*Gr39b*	933	-	-
*Or45b*	1179 *	-	-	*Gr43a*	441	-	-
*Or46a*	1158 *	-	-	*Gr47b*	1101	-	-
*Or47a*	1164	-	-	*Gr57a*	1245 *	Yes (*p* = 0.0000)	6 (*p* < 0.0326)
*Or47b*	945	-	-	*Gr58b*	696	-	-
*Or49a*	1212	-	-	*Gr58c*	1071	-	-
*Or49b*	1113 *	-	-	*Gr59b*	459	-	-
*Or56a*	1101 *	-	-	*Gr59d*	1023	-	-
*Or59a*	981	-	-	*Gr59e*	864	-	-
*Or59b*	1194 *	-	-	*Gr59f*	1152	-	-
*Or63a*	1266 *	Yes (*p* = 0.0000)	17 (*p* < 0.0446)	*Gr61a*	1122	-	-
*Or67a*	1212 *	-	-	*Gr63a*	1428 *	-	-
*Or67b*	1032 *	-	-	*Gr64a*	1191	-	-
*Or67c*	1212 *	-	-	*Gr64b*	831	-	-
*Or67d*	1170	-	-	*Gr64c*	999	-	-
*Or69a*	1143 *	-	-	*Gr64e*	1071	Yes *(p* = 0.040)	1 (*p* = 0.0457)
*Or71a*	1107 *	-	-	*Gr64f*	1059	-	-
*Or74a*	1215 *	-	-	*Gr66a*	753	-	-
*Or82a*	1101 *	-	-	*Gr68a*	703	-	-
*Or83a*	1320 *	-	-	*Gr77a*	1257 *	Yes (*p* = 0.0000)	4 (*p* < 0.0415)
*Or83c*	999	-	-	*Gr89a*	1095 *	-	-
*Or85b*	496	-	-	*Gr93a*	1299	-	-
*Or85c*	1110	-	-	*Gr93c*	1170 *	-	-
*Or85d*	564	-	-	*Gr94a*	1185 *	-	-
*Or85e*	1326	-	-	*Gr97a*	1207	-	-
*Or85f*	1203	-	-	*Gr98a*	855	-	-
*Or88a*	1203	-	-	*Gr98b*	747	-	-
*Or92a*	807	-	-	-	-	-	-
*Or94a*	1164	-	-	-	-	-	-
*Or98b*	927	-	-	-	-	-	-
*Orco*	1029	-	-	-	-	-	-

Note - The * symbol after the size number indicates a complete CDS recovered. Blank values on the selection tests columns correspond to genes for which we could not mount genes matrices due to the quality of the sequences. Genes with no significant signal of positive selection present the dash signal (-) on the selection tests columns.

Notwithstanding, results for the other *Drosophila* species were based on alternative methodologies, the number of chemoreceptor genes associated with ORs genes varied from 38 (in *D. busckii*) to 60 (in *D. melanogaster, D. sechellia* and *D. simulans*) (Table 1, Table S5). GRs varied from minimum values of 34 (in *D. busckii*) to maximum values of 60 (in *D. melanogaster, D. sechellia,* and *D. simulans*). 

**Figure 3 -:**
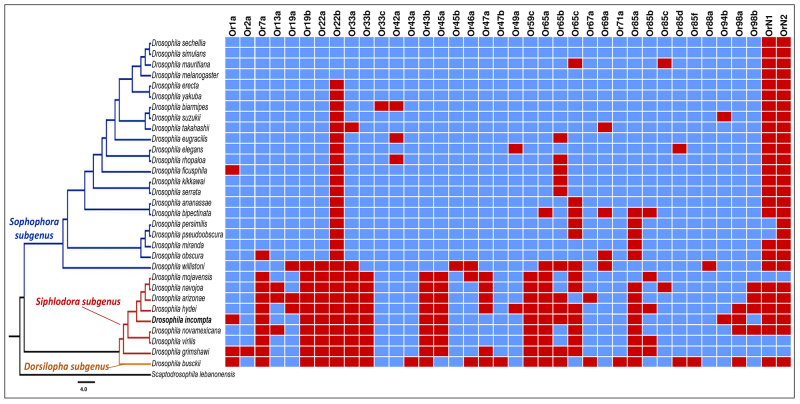
Presence (blue boxes) and absence (red boxes) of 38 olfactory genes (****OR*)*** analyzed across the *Drosophila* phylogeny, modified from Robe et al. (2010), Yang et al. (2012), Yassin (2013), Hjelmen and Johnston (2017), and O’Grady and DeSalle (2018). Orthologues that were found in all analyzed species were not depicted on this graph.

**Figure 4 -:**
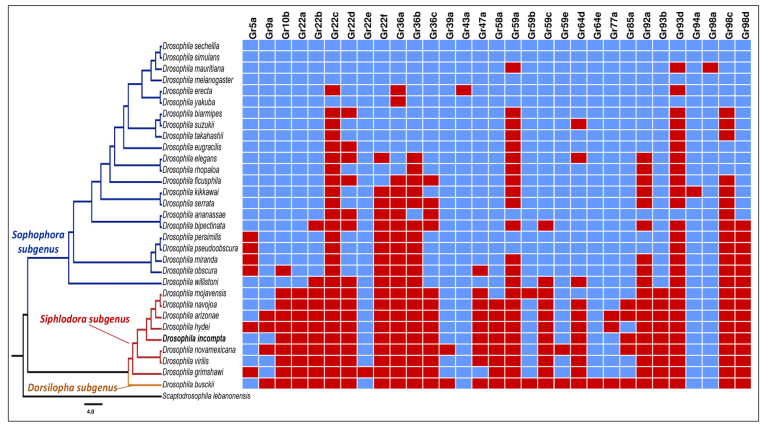
Presence (blue boxes) and absence (red boxes) of 31 gustatory genes (***GR*** ) analyzed across the *Drosophila* phylogeny, modified from Robe et al. (2010), Yang et al. (2012), Yassin (2013), Hjelmen and Johnston (2017), and O’Grady and DeSalle (2018). Orthologs that were found in all analyzed species were not depicted on this graph.

Regarding the distribution of gene losses or gains, evidence of phylogenetic signal was found for both ORs and GRs (Figures 3 and 4). In this context, it was interesting to see that the number of sequences found for both GR and OR was generally lower in non-*Sophophora* species. Nevertheless, two specific gene losses that are either autapomorphic or homoplastic for *D. incompta* were found for the genes ORs *Or1a,* and *Or94b* (Fig. 3).

### Selection tests

Of the 84 chemoreceptor genes found for *D. incompta*, 81 had a continuous coding sequence of at least 441 bp (147 aa) and were included in further evolutionary analysis. Three ORs were not included due to their fragmentary sequences.

Of the 81 chemoreceptors matrices used for selection tests, six presented signals of positive selection on the aBSREL approach (Table 2): OR19a, OR42a, OR63a, GR57a, GR64e and GR77a (*p* < 0.05). These six matrices were also tested on FEL analysis and presented signals of positive selection ranging from one site for GR64e to 17 sites for GR63a, according to the following distribution: four sites for GR77a, five sites for OR19a, six sites for GR57a and eight sites for OR42a. 

### Discussion

*Drosophila* has been widely used as a model to study the forces shaping the evolution of chemoreceptor gene families in different ecological contexts. Nevertheless, the role that selection and genetic drift have played in the evolution of these genes in specialized species is still controversial (McBride, 2007; McBride and Arguello, 2007; Gardiner et al., 2008). This study contributes to the understanding of the patterns of molecular evolution exhibited by the OR and GR gene families in a species with a strict niche of the *D. flavopilosa* group, *D. incompta*, and provides several insights into understanding the general patterns of the entire *Drosophila* genus.

### Molecular evolution of OR and GR gene families in *D. incompta*

Although it has been previously demonstrated that *D. incompta* can only explore *Cestrum* flowers as feeding and breeding resources (Sepel et al., 2000; Santos and Vilela, 2005; Robe et al., 2013), the ethological or physiological characteristics associated with this restricted ecology have not yet been recognized. Our results regarding the olfactory preferences of *D. incompta* suggest a chemosensory basis for this condition, as indicated by the clear preference for extracts of *Cestrum* flowers compared to other scents (*Brunfelsia*, banana, or orange extract scents). It is generally expected that at least part of this specialization is related to chemosensory changes, ultimately linked to genetic alterations. This hypothesis is supported by some of the results presented here for the OR and GR gene families in *D. incompta*, such as some autapomorphic or homoplastic gene losses suggested for some genes, in contrast to the signals of positive selection detected for others. 

It is well known that both OR and GR gene families frequently evolve through gene duplication, gene loss, and pseudogenization (Gardiner et al., 2008; Benton, 2015). In particular, it has been suggested that during host specialization there may be a general trend toward loss of some chemosensory genes with concomitant positive selection on others (McBride, 2007; McBride and Arguello, 2007). For *D. incompta*, orthologous sequences were found for 84 of the 120 chemoreceptor genes examined in this study. Different strategies were employed for this task, among which InsectOR revealed the most sensitive, being able to recover 42 of the 44 ORs, and all the GRs, through the use of a mixed query matrix containing *D. melanogaster* and *D. virlis* chemoreceptor genes. Consistency of these results were further revealed by the ML tree reconstructed with amino acid sequences for each family, where most identifications performed with InsectOR recovered monophyletic groups. In fact, the only exceptions to this pattern were related to polytomous or poorly supported relationships, in which putatively orthologous sequences encompassed short branches that were located as adjacent in the three. Such results are probably an outcome related to the insufficient phylogenetic signal presented by some sequences, or to the positive selection detected for others (see below), since none *D. incompta* sequence appeared as nested within clades compounded by alternative orthogroups.

Although the small number of genes recovered for *D. incompta* seems to be consistent with the hypothesis suggesting a general trend of gene loss in specialized species (McBride and Arguello, 2007; Croset et al., 2010), this pattern should be interpreted with caution. At first, it is important to consider that at least some of these putative losses could be an outcome of methodological sources of error related to sequencing coverage, genome assembling or annotation. Moreover, a closer inspection to the general phylogenetic profile reveals that most of the missing chemoreceptor genes detected in *D. incompta* appear to have been lost in the ancestors (see below), reflecting a phylogenetic signal or a historical contingency. Indeed, only two of the 36 missing genes of *D. incompta* provided evidence of autapomorphic or homoplastic gene loss in the target species. This is true, for example, for the putative loss of *OR1a* and *OR94b* in the *D. incompta* genome, which were previously shown to respond to specific odorants, like the aldehyde (E)-2-hexenal (Fishilevich et al., 2005) and the phenol 4-methylphenol (Kreher et al., 2005), respectively. Interestingly, neither of these compounds seem to have been isolated in *Cestrum* yet (Alrabayah et al., 2024).

The effects of differential selection pressure in the repertoire of chemoreceptor genes of *D. incompta* could be directly tested in the positive selection tests, when tree genes of both the GR and the OR gene families showed signals of positive selection. Interestingly, two of the six genes exhibiting such signals (OR63a and GR57a) were found in all other species examined, suggesting that their products serve some important functions that are somehow conserved across the *Drosophila* phylogeny. Otherwise, GR64e seems to be missed only in *D. busckii*, and was previously shown to confer responsiveness to glycerol, a byproduct of yeast fermentation, affecting feeding preferences in *Drosophila* (Wisotsky et al., 2011). Since yeasts are common inhabitants of many angiosperm nectars (Pozo et al., 2009), this result raises the possibility that yeasts are the effective feeding resources used by *D. incompta* larvae and/or adults in *Cestrum* flowers, suggesting that the *D. flavopilosa* group may not have diverged so much from the ancestral Drosophilidae feeding habits (Throckmorton, 1975).

### Evolution of OR and GR genes in *Drosophila*

By increasing the number of species studied, we improved our understanding of the evolutionary patterns of the OR and GR gene families across the phylogeny of *Drosophila*. In this sense, we found a large variation in the number of chemoreceptor genes among species, with the higher and lower numbers of OR and GR genes generally found for species of the subgenus *Sophophora*, especially for species of the *melanogaster* subgroup, and for *D. busckii,* respectively. Because *D. melanogaster* was one of the species used as a query or seed in our searches, the higher number of chemoreceptor genes found for this species and other closely related taxa may be a methodological artifact. The lower number of orthologous sequences found for *D. busckii* could also be a biased result, as this species is not closely related to either of the two species used as a query or seed in our searches (Yassin, 2013; O’Grady and DeSalle, 2018). Moreover, chemoreceptor annotation for this species has not yet received special attention. Nevertheless, such a pattern does not explain the results found for species closely related to *D. virilis*, since the last species not only presents a well-annotated genome but was also used as a query or seed in our searches. Even so, the number of orthologous sequences found in this species is close to the lower range for the OR and GR genes (50 and 40, respectively). Another point to consider is that we are only looking at one copy of each gene, which means that the scenario presented focuses on more ancient events of gene duplication. In this sense, the number of genes may be somewhat underestimated for some species. This is the case for *D. grimshawi,* which has already been shown to have multiple copies of some loci associated with recent gene duplications (Guo and Kim, 2007; Nozawa and Nei, 2007; Gardiner et al., 2008). Even so, it is important to mention that our OR family annotation generally agrees with Ramasamy et al. (2016) concerning the number of genes for each species. The few divergences may be explained by the use of different annotation tools, albeit the main approach of both studies was the BLAST analyses. 

**Figure 5 -:**
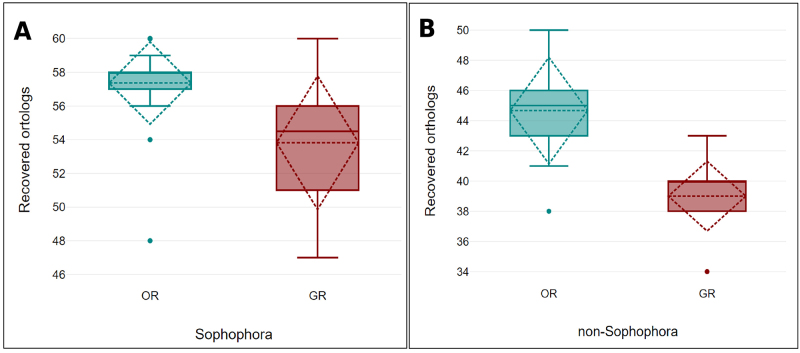
Box plot graph comparing the total number of orthologs found for each of the two gene families by different *Drosophila* lineages (**A**: *Sophophora* species and **B**: non-*Sophophora* species). The bold horizontal line in the boxes represents the median, while the dashed lines represent the mean with standard deviation. Dots represent the outliers.

Despite some putative sources of bias, the general picture suggests that the distribution of OR and GR genes across the *Drosophila* phylogeny may reflect a phylogenetic signal related to gene gains or losses in ancestral populations that have been stably inherited over millions of years. These conclusions are further supported when the presence or absence of each of the 16 OR and 20 GR genes of *D. melanogaster* not found in *D. incompta* are evaluated in a phylogenetic context. Of the ORs not found in *D. incompta,* six were also not found in any non-*Sophophora* species (OR7a, OR33a, OR33b, OR43b, OR59c, and OR65a), and three were found in only one species of the subgenus *Siphlodora* (OR65c in *D. arizonae*, and OR85a in *D. mojavensis*) or the subgenus *Dorsilopha* (OR45a in *D. busckii)*. This pattern also holds for the gene family GR, where most genes not found in *D. incompta* appear to be largely restricted to the *Sophophora* subgenus. Indeed, 16 GR genes (GR10b, GR22a, GB22b, GR22c, GR22d, GR22f, GR36a, GR36b, GR36c, GR59a, GR59c, GR92a, GR93b, GR93d, GR98c, and GR98d) were not found in any non-*Sophophora* species, and two of them (GR22c and GR93d) were present in only five *Sophophora* species. These absences may indicate ancestral gene losses in non-*Sophophora* species, gene gains in *Sophophora* species, or even high divergence compared to query sequences reaching the limit of sensitivity of BLAST. In fact, comparisons involving the mean number of chemoreceptor genes between *Sophophora* with 57.36 (σ = 2.44) for *OR* and 53.82 (σ = 3.96) for *GR* genes, and non-*Sophophora* species with 44.67 (σ = 3.53) for OR and 39 (σ = 2.31) for GR genes, further agrees with these putative phylogenetic trends (Figure 5). Nevertheless, there are interesting exceptions to this rule, like *D. willistoni* and *D. busckii*, which stands out as a *Sophophora* and a non-*Sophophora* species with a lower number of OR and GR genes respectively.

Because species in the *melanogaster* group often have the greater number of OR and GR genes (60 for both in *D. melanogaster*), it is tempting to speculate that, in at least some cases, the number of chemoreceptor genes also defines evolutionary potentials related not only to niche breadth but also to the distribution range of individual species within each of the major lineages of *Drosophila.* Indeed, several *Sophophora* species are recognized as generalists and cosmopolitans, such as *D. ananassae* (Singh, 1996), *D. kikkawai* (Ramniwas and Kajla, 2012), *D. mauritiana* (Tsacas and David, 1974), *D. melanogaster* (David and Capy, 1988; Lachaise and Silvain, 2004; Markow, 2015), *D. obscura* (Begon, 1975; Markow and O’Grady, 2008), *D. persimilis* (Wogaman and Seiger, 1983), *D. pseudoobscura* (Carson, 1971), *D. serrata* (Kelemen and Moritz, 1999; Schiffer and McEvey, 2006), *D. simulans* (Lemeunier et al., 1986), *D. suzukii* (Asplen et al., 2015; Biondi et al., 2016), and *D. willistoni* (Spassky et al., 1971, Cordeiro and Winge, 1995; Powell, 1997), a pattern that appears to be less common in non-*Sophophora* species. Nevertheless, this hypothesis also has some important exceptions, as in the case of *D. sechellia*, a *Sophophora* species with 120 chemoreceptor genes which is endemic to the Seychelles islands in the Indian Ocean and is specialized to a single resource, the fruit of *Morinda citrifolia* (Tsacas and Bächli, 1981; Louis and David, 1986; Gerlach, 2009); and *D. erecta,* another species of this subgenus, which has 59 and 56 OR and GR genes, respectively, and is endemic to west-central Africa, and specialized on ripe fruit of *Pandanus* spp. (Lachaise and Tsacas, 1974; Rio et al., 2010; Linz et al., 2013). This reinforces that, although several trends and potentials seem to have been inherited from ancestral populations, the patterns and processes behind the evolution of OR and GR gene families are certainly quite complex and need to be evaluated under different perspectives.

## Conclusion

The evolution of specialization in the use of specific resources is an interesting topic that is not yet fully understood (Etges, 2019; Markow, 2019; Auer et al., 2020). Here, we demonstrated the clear preference of *D. incompta* for the scent of macerated *Cestrum* flowers compared to other resources and characterized some genomic changes on the OR and GR genes that are possibly related to this behavior. In characterizing the chemoreceptor repertoire of *D. incompta*, we not only detected some autapomorphic or homoplastic gene losses, but also found several indications of positive selection on different components of the two major gene families that encompass some of the basic tools for interacting with nature (Benton, 2015). However, most gene losses in this species appear to have first occurred in its ancestors located at different nodes of the *Drosophila* phylogenetic tree. So, generally speaking, our results do not seem to corroborate the idea that specialized species have a more limited number of ORs and GRs. Nevertheless, the precise relationship between these changes and the ecological potential of each species remains largely unclear, and it is not currently possible to decipher the putative causes and consequences of ecological specialization. 

## Supplementary Material

The following online material is available for this article:

Figure S1 - Phylogenetic tree reconstructed with amino acid sequences related to the odorant receptor (OR) gene family in *D. incompta*, *D. melanogaster* and *D. virilis*.

Figure S2 - Phylogenetic tree reconstructed with amino acid sequences related to the gustatory receptor (GR) gene family in *D. incompta*, *D. melanogaster* and *D. virilis*.

Table S1 - Sample design of the five olfactory choice tests performed with *Drosophila incompta* (A, B, and C) and *D. melanogaster* (D and E), comparing odors of *Cestrum* × Banana (replicates A and D), *Cestrum* × Orange (replicates B), *Cestrum* × *Brunfelsia uniflora* (replicates C), and Banana × Orange (replicates E).

Table S2 - Metrics and statistics of *Drosophila incompta* genome and transcriptome draft assemblies.

Table S3 - Accession numbers (GenBank - NCBI) of the sequences used in evolutionary analysis matrices.

Table S4 - Olfactory (OR, sheet A) and gustatory receptor (GR, sheet B) orthologues recovered for *Drosophila incompta* by each tool.

Table S5 - List of presence (X on the table) or absence (- on the table) of olfactory (OR, sheet A) and gustatory receptor (GR, sheet B) orthologues retrieved for each of the analyzed *Drosophila* species.
