# Supplementary material for: Characterization of the chemoreceptor repertoire of a highly specialized fly with comparisons to other Drosophila species
Source: Genet Mol Biol. 2024 Jun 14;47(2):e20220383. doi: 10.1590/1678-4685-GMB-2022-0383 (PMC11182316; doi:10.1590/1678-4685-GMB-2022-0383)
Supplement: Figure S2 - [file 1415-4757-GMB-47-2-e20220383-s2.pdf]

Genetics and Molecular Biology
https://doi.org/10.1590/1678-4685-GMB-2022-0383

Supplementary Material to: "Characterization of the chemoreceptor repertoire of a highly specialized fly with comparisons to other *Drosophila* species"

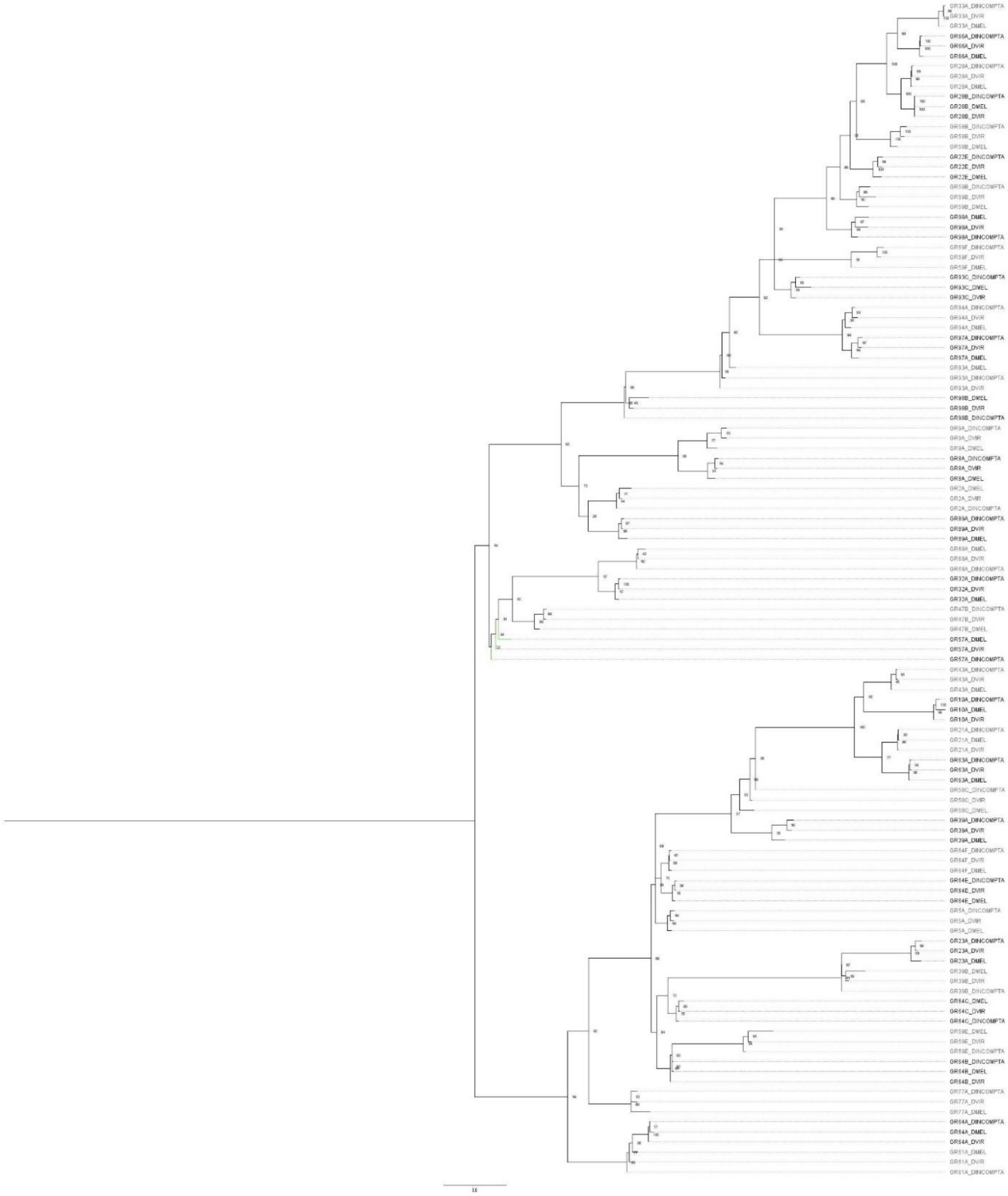

Figure S2 - Phylogenetic tree reconstructed with amino acid sequences related to the gustatory receptor (GR) gene family in *D. incompta*, *D. melanogaster* and *D. virilis*. Numbers near the internal nodes reflect bootstrap support. Branches representing individual genes which did not recover a single clade in the three are colored.
